# Seasonal and temporal changes during storage affect quality attributes of green asparagus

**DOI:** 10.1016/j.postharvbio.2019.111017

**Published:** 2020-01

**Authors:** Maria Anastasiadi, Emma R. Collings, Allan Shivembe, Binghua Qian, Leon A. Terry

**Affiliations:** a Plant Science Laboratory, Cranfield University, Bedfordshire, MK43 0AL, UK; b Cobrey Farms, Ross-on-Wye, Herefordshire, HR9 5SG, UK

**Keywords:** Sugars, Cultivar, Storage, Abscisic acid (ABA)

## Abstract

Asparagus is a perennial crop with a short UK harvest season. Methods to extend the storage life of asparagus have proven difficult. To gain insight into the physiological (*viz*. colour, respiration rate, cutting energy, and stiffness measured using laser Doppler vibrometry), and biochemical (*viz*. sugars, ascorbic acid, and abscisic acid and its catabolites) changes throughout the UK season, two cultivars were harvested weekly and stored under shelf life conditions (7 °C). Results were compared to spears (plus one additional cultivar) cold stored (1 °C) for three weeks followed by one week of shelf life. Concentrations of sugar, abscisic acid (ABA) and catabolites at harvest were subject to seasonal variation, directly affecting storage potential. A generalised linear model with stepwise feature selection was applied to select the most important parameters for the prediction of total sugars and phaseic acid (PA). More favourable growing conditions at harvest increased sugars and lowered ABA content and catabolites, which coincided with better maintenance of spear quality during storage; including maintaining textural characteristics. Storage time had a negative impact on spear texture and sugar content, with cutting energy increasing and stiffness decreasing both during cold storage and subsequent shelf life. A partial shift in sugar biosynthesis occurred during shelf life increasing sucrose concentrations. Results suggest that the temporal flux in ABA and catabolites, and individual sugars could be used to model storage potential of asparagus spears.

## Highlights

• Favourable conditions at harvest lowered ABA and increased total sugar content.
• Low ABA in spears at harvest reduced rate of senescence during storage.
• Shift in sugar metabolism associated with changes in light and temperature.
• Storage time had a negative impact on spear texture and sugar content.

## 1 Introduction

The UK asparagus (*Asparagus officinalis*) season is relatively short and typically lasts from April to June, with a late harvest in September accomplished by using a reverse season technique (Hart, 2015). Extension of the main UK season has mainly been achieved using early and late maturing cultivars such as ‘Gijnlim’ and ‘Guelph Millennium’, respectively (Rowland, 2009).

Asparagus is among the most perishable postharvest commodities primarily due to its high metabolic rate, which can lead to accelerated senescence processes and limited postharvest life (Mastropasqua et al., 2016). The appearance of the spear (shape, size, girth, and colour) is considered the most critical quality attribute for commercial grading standards; whereas organoleptic attributes (texture, taste, aroma, flavour and bitterness) are important features used by consumers to assess quality (Siomos, 2018). During air storage, the deterioration of asparagus is accompanied by rapid sugar loss, colour changes due to chlorophyll breakdown, undesirable texture changes (lignification), ascorbic acid and organic acid loss, and asparagine accumulation (Renquist et al., 2005; Albanese et al., 2007; Verlinden et al., 2014). Commercial practices in the UK involve a ‘stock rotation’ system where asparagus spears are stored at 2 °C for five days or more prior to distribution to suppliers/consumers, which includes a further seven days under shelf life conditions (at *ca*. 7 °C) (personal communication from Cobrey Farms). Cold storage has been reported to maintain lower respiration rate and suppress the catabolism of quality-related compounds (Lipton, 1990), but it can promote lignification (Toscano et al., 2018). Similarly, the role of ABA and its catabolites during asparagus storage has not been investigated. During spear elongation in the field, spear tips have been reported to maintain high concentrations of ABA compared to other parts of the spear (Kojima et al., 1993), but to date there is little understanding of how ABA concentrations change during the harvest season and the potential impact this may have on asparagus storability.

Bhowmik et al. (2002) detailed a decline in soluble sugars and organic acid content in freshly harvested spears during the season with losses highest in the top region of the spear. Subsequent shelf life was also reduced by 50%. Furthermore, changes in temperature/climatic conditions had a significant impact on texture and biochemical profile of spears (Bhowmik et al., 2002; Shou et al., 2007). Spears grown in the UK are harvested for 6–8 weeks between April–June, and are cultivated in polytunnels (early season) or open field. Upon completion of the harvest season, spears are allowed to produce ferns for the remainder of the summer to replenish carbohydrate reserves in the roots (Siomos, 2018).

To date, limited research has assessed how seasonal variation affects asparagus physiology and biochemistry during postharvest storage. The aim of this study was therefore to determine how quality parameters measured at harvest and during shelf life were influenced by seasonal variation (over five weeks) and spear diameter (grade) in cultivars commonly grown in the UK. To our knowledge, this is the first study to consider the influence of spear grade on storability.

## 2 Materials and methods

### 2.1 Chemicals

All HPLC and LC–MS grade solvents were obtained from Fisher Scientific (Loughborough, UK). Metaphosphoric acid (Bioxtra ≥ 33.5%), l(–) – ascorbic acid and d-fructose were obtained from Sigma-Aldrich (Dorset, UK). d-glucose and sucrose were purchased from Fisher Scientific (Loughborough, UK). Deuterated labelled and unlabelled compounds, [2H3]-dihydrophaseic acid (−)-7′,7′,7′-d3 dihydrophaseic acid (DPA); [2H3]-phaseic acid (−)-7′,7′,7′,-d3-PA, [2H4]-7′-hydroxy-abscisic acid ( ± )-5,8′,8′,8′-d4-7′-hydroxy-ABA; [2H4]-abscisic acid (−)-5,8′8′8′-d4-ABA, [2H6]-abscisic acid ( ± )-3′,5′,5′,7′,7′,7′-d6-ABA; (−)-DPA, (−)-PA, ( ± )-7′-hydroxy-ABA were obtained from the National Research Council of Canada-Plant Biotechnology Institute; (±)-ABA was purchased from Sigma-Aldrich.

### 2.2 Plant material for seasonal variation

Two green asparagus cultivars supplied by Cobrey Farms, a commercial farm in Herefordshire, UK (52°05′N, 2°45′W) were selected to study spear quality attributes during storage and assess the impact of seasonal variation. Spears were harvested on a weekly basis (from the same field) for five weeks in total from 25th May to 23rd June 2016. The cultivars included “Gijnlim’ F1′, an early high yielding all male hybrid developed in The Netherlands representing more than 70% of UK asparagus production, and ‘Guelph Millennium F1′, a late maturing all male hybrid developed in Canada and adapted for cooler areas. Plants were grown on sandy soil under commercial growing conditions. Cultivars ‘Gijnlim’ and ‘Guelph M.’ crowns were 13 and 7 years old at the time of harvest, respectively. At harvest, spears were hand-cut at ground level between 0730 and 0900 h at field temperatures of 12 ± 4 °C and hydro-cooled within 2 h from harvest. After hydro-cooling spears ranging between 10 to 20 mm in diameter were sorted by hand according to grade into small [12–13.9 mm], medium [14–16.9 mm] and large grade [17–20 mm] as is standard commercial practice. Asparagus were stored overnight at 2 °C and transported under refrigeration (at 4 °C) to Cranfield University the next day within 3 h. Upon arrival, spears were trimmed to 15 cm length and packaged separately in trays wrapped with commercial polyvinyl chloride (12.7 μm thick) film (perforated with nine small holes [*ca.* 1 mm diameter]). Samples were prepared in triplicate and subsequently stored at 7 °C in an environmental test chamber (Sanyo MLR-351H, Osaka, Japan) under artificial light (*ca*. 1,358 lx) and 70% RH for five days to simulate retail conditions. Three trays per grade and cultivar were sampled at regular intervals (*viz*. 0, 2 and 5 d) for physiological and biochemical analysis.

### 2.3 Plant material for cold storage trial

For cold storage assessment, the same two cultivars plus ‘Jaleo’ a clonal hybrid from Vilmorin Seed Co., adaptable to warm climates were selected. Medium spears from all three cultivars were harvested on 25th May 2016 and processed at Cobrey as described in Section 2.2. The selected asparagus spears were transported to the laboratory the following day by refrigerated transport (at 4 °C). Upon arrival in the laboratory, approximately 700 g (*ca*. 30 spears) of each cultivar were randomly divided into three sealed polypropylene boxes (12 L) containing a freshness tray (allowing air to circulate underneath produce) and the spears were stored at 1 °C for three weeks, in the absence of light. Each box contained a 100 mL conical flask filled with water. A plastic tube was inserted into the water and connected to an air supply (flow rate: 250–300 mL min^−1^) using an ICA gas mixing system (Storage Control Systems Ltd, Kent, UK) to maintain relative humidity above 95% and avoid CO_2_ accumulation. At the end of the storage period, six asparagus spears per cultivar were trimmed to 15 cm length and packaged in trays wrapped with commercial polyvinyl chloride (12.7 μm thick) film (perforated with 9 small holes [*ca*. 1 mm diameter]) in triplicate. The trays were subsequently stored at 7 °C for seven days in an environmental test chamber under the same conditions described in Section 2.2. The RH and temperature in the boxes were continuously monitored using Gemini Tiny Tag Ultra 2 data loggers (−95% RH, −25 to 85 °C temperature, Part No; TGU-4500). Sampling for physiological measurements and biochemical analysis was done at weekly intervals from every box during the storage period at 1 °C (day 7, 14, 21) and from the trays at the end of shelf life at 7 °C (day 28). Prior to sampling, the respiration rate was measured from each storage box by passing air directly to a Sable Respirometry system as described in Collings et al. (2019), with slight modifications. Measurements were obtained using a higher flow rate of 250–300 mL min^−1^. Sampled air was analysed over a 2 min period, three times (cycles), to obtain one average measurement per day.

### 2.4 Sampling and quality assessment

Upon arrival (day 0) and at each sampling point, six spears per grade (n = 18) were randomly selected from each cultivar and subjected to colour and textural evaluation (stiffness, cutting energy) as described in Sections 2.5–2.7. After quality assessment, spears were sectioned at 4 and 11 cm from the tip for medium and large grade and at 3 and 11 cm for small grade, and the apical and mid-sections of each spear were immersed into liquid nitrogen. The snap-frozen samples were stored at −40 °C until analysis. Prior to extraction, the plant material was freeze-dried and powdered in a homogenizer (Precellys 24, Stretton Scientific Ltd, UK) at 5000 rpm for 20 s using ceramic beads. The freeze-dried powder was stored at −40 °C.

### 2.5 Colour measurement

Asparagus spears were trimmed to 15 cm and marked at 4 cm and 11 cm from the top to separate into three sections: tip, mid and base. Samples were placed inside a Photo-E-Box plus 1419 set at D65 (6500 K) under LED lights (back, left and right) and images of the whole spear were captured using a Lumenera Infinity 3 high definition digital camera with CCD colour sensor (Lumenera Corporation, Ottawa, ON). Objective colour measurements were extracted and processed with the associated Infinity Analyse software version 6.5 to obtain colour parameters (red [R*], green/red axis component [G*] and yellow/blue axis component [B*]) for each selected region (*viz*. tip, mid and base). The RGB values were later converted into lightness (L*), chroma (C*) and hue angle (h°) using standard RGB conversion equations for D65 (Easy RGB, 2018). Extracting colour data from these images had the advantage of allowing specific regions along the spear to be assessed and from a larger region, providing a more representative data set compared to traditional colorimeter measurements. By using global colour images, certain areas such as bracts or damaged regions could also be excluded to avoid skewing the data.

### 2.6 Stiffness measurement

Stiffness was evaluated using a laser Doppler vibrometer (LDV) Polytec PDV 100 as described by Landahl and Terry (2012). A piezoelectric sensor hammer was connected to the integrated circuit piezoelectric sensor (ICP) signal conditioner. The mechanical strain was converted into an electrical signal and amplified using a Polytec VIB-E-220 data acquisition system. The signal was then analysed using the Polytec vibrometer software. This showed the peaks generated from the vibration of the asparagus measured in terms of frequency (kHz) (at maximum peak). Stiffness was then calculated by dividing frequency with the mass (kg) of each spear.

### 2.7 Cutting energy measurement

Cutting energy was measured with a uniaxial testing machine (Instron 5542, Instron, Buckinghamshire, UK) as described by Landahl et al. (2009) with modifications. A 0.25 mm thick cutting blade with a load cell calibrated at 500 N was used at 600 mm/min cross head speed and 6 mm penetration depth. Cutting energy (mJ) defined as the force required for cutting the spear between a depth of 2 and 4 mm was used for textural evaluation at two different positions: apical (4 cm from the tip) and middle (11 cm from the tip).

### 2.8 Soluble sugars

Extraction and analysis of fructose, glucose and sucrose was performed as previously described (Anastasiadi et al., 2016). Prior to analysis, the sugars extracts were diluted 1:1 v/v and 1:3 v/v with HPLC grade water for tip and mid extracts respectively. The eluted compounds were detected by Evaporative Light Scattering Detector (ELSD) and quantification was based on external calibration curves of commercial standards.

### 2.9 Total ascorbic acid

Changes in total ascorbic acid over the season and during cold storage was determined as previously described (Anastasiadi et al., 2016) with slight modifications. Briefly, 100 mg of freeze-dried asparagus powder from apical and mid sections were mixed with 2.5 mL of degassed metaphosphoric acid solution (0.01 mol L^−1^) and the mixture extracted in a shaking water bath at 25 °C for 10 min. For seasonal variation, only the mid-section was extracted due to the lack of adequate material from the tip section, especially for the small grade spears. The extract was filtered through 0.2 μm nylon filters and immediately injected in the HPLC. Separation was achieved with a ZORBAX Eclipse XDB-C18 (5 μm, 250 mm × 4.6 mm) column. The mobile phase consisted of 0.01 M ammonium acetate. An isocratic elution system was used at a flow rate of 0.7 mL min^−1^ with 10 min duration. Ascorbic acid was monitored at 248 nm and quantified using an external calibration curve of l-(–)-ascorbic acid. The assay was performed in triplicate.

### 2.10 ABA and catabolites

The seasonal variation in ABA and ABA catabolites were assessed according to the method by Ordaz-Ortiz et al. (2015) with modifications. Briefly, 3 mL of methanol/water/formic acid mixture (75:20:5, v/v), containing 25 ng mL^−1^ internal standard mix, was added in a 15 mL falcon tube containing 50 mg of freeze-dried medium grade asparagus material (tip and middle sections). The tubes were put in a Multi Reax tube shaker (Heidolph Instruments GmbH & Co, Germany), placed in a dark chamber set to 4 °C and were extracted for 1 h at 1000 rpm. The tubes were centrifuged for 10 min at 4000 rpm at 4 °C, and the supernatant was transferred into clean tubes and subsequently freeze-dried overnight in the absence of light. The extracts were reconstituted with 400 μL of water/acetonitrile/formic acid mixture (89.9/10/0.1 v/v). The samples were immediately injected on an Agilent 1290 Infinity UHPLC system coupled with an Agilent 6540 Ultra High Definition Accurate Mass Q-TOF LC–MS System (Agilent Technologies) and quantified as described by Ordaz-Ortiz et al (2015).

### 2.11 Statistical assessment

Statistical analyses were carried out using Statistica for Windows version 10, 64-bit (Statistica Tulsa, OK 74104, USA). Analysis of variance (ANOVA) was used to identify significant differences (P < 0.05) between treatments followed by Tukey’s post-hoc test. Standard deviation (SD) for each mean are displayed in each applicable figure and table, and represents the standard error estimated from the residual mean square. The R package caret was used for regression analysis. A generalised linear method with stepwise feature selection (lmStepAIC) was employed to build a regression model predicting sugar and phytohormone content in the tip of both cultivars using average weekly values for each weather variable considered before each harvest date. This method employs feature selection in a stepwise manner; therefore, the final model only contains the most important variables for the prediction. The models were developed using 70% of samples as a training set and evaluated using an independent test set comprising 30% of the samples.

## 3 Results

### 3.1 Physiological and biochemical changes during shelf life over the course of the season

The UK season was found to have a limited effect on asparagus colour where L* and C* remained stable with no significant differences between cultivar or harvest week, with the exception of C* in the tip, which showed some minor fluctuation over the course of the season in both cultivars. Similarly, hue angle fluctuated between weeks, the main difference being noted at week one where hue angle was significantly lower (less green) in all spatial regions (Supplementary material 1). Grade of spear did not influence colour.

Cutting energy tended to increase towards the end of shelf life in samples of both cultivars, but the observed increase was most pronounced on the second week of harvest (Supplementary material 2). There were no significant differences between different grades of spears or spatial regions. In contrast, there were seasonal differences observed for cutting energy between spatial regions with values initially higher in tips compared to the mid-sections, but this trend was reversed from week 3 onwards (Supplementary material 3a). Similarly, stiffness decreased during the season, irrespective of cultivar (Supplementary material 3b); except at week one where ‘Guelph M.’ had higher values (0.0025 kHz kg^−1^) compared to ‘Gijnlim’ (0.0018 kHz kg^−1^). With respect to grade, large spears were stiffer at the beginning of the season (*ca*. 0.003) compared to medium and small grades (*ca*. 0.018). The same trend was also observed during shelf life with stiffness values for larger spears being overall 1.5-fold higher compared to medium and 2.8-fold higher compared to small (data not shown).

Spear size also seemed to influence moisture loss, with small spears (larger surface area to volume ratio) showing an average moisture loss of 6.2% throughout shelf life followed by medium (3.85%) and large grade (3.3%) (smaller surface area to volume ratio). Furthermore, moisture loss was greatest in small spears stored towards the end of the season (when spear diameters are at their smallest) especially in ‘Guelph M.’ (up to 10–13%) (Supplementary material 4). Moisture loss during shelf life in spears of the other two grades assessed increased with duration but was not as extreme. In contrast, spears harvested earlier in the season had significantly lower moisture loss (up to 5%). Spears harvested on week three had overall the lowest amount of water loss (3.48%) while spears harvested on week five had the highest loss (5.73%) (Supplementary material 4).

**Fig. 1:**
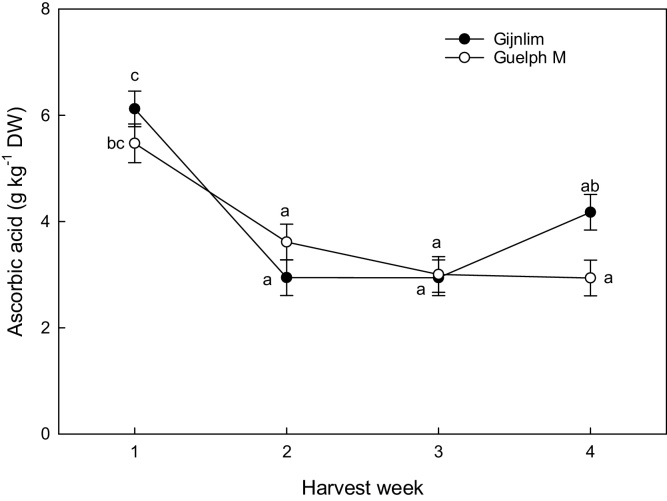
Seasonal changes in ascorbic acid (g kg^−1^ DW) (ascorbic acid) (mid-section) in two UK grown asparagus cultivars (‘Gijnlim’ and ‘Guelph Millennium’) measured across four consecutive harvest weeks. Data represent a mean of three grades per cultivar (n = 9)  ±  standard deviation (SD). Different letters denote significant differences (for interaction between week*cultivar).

Apart from physiological traits, seasonal variation and storage conditions also had a significant impact on compounds related to both nutritional value and organoleptic characteristics of asparagus spears. Ascorbic acid content was measured in the mid regions of asparagus spears and was found to decline early on during the season (week 1 to 2) with values dropping from 6 to 3 mg g^−1^ DW (Fig. 1). Subsequently, spears harvested at week one and two maintained significantly higher ascorbic acid concentrations (5.3 g kg^−1^ DW and 6.5 g kg^−1^ DW respectively) after three days of shelf life compared to week three (3.8 g kg^−1^ DW).

Sugar profile followed a spatial distribution with fructose and glucose being the dominant sugars present in mid regions followed by sucrose. In contrast, sucrose and fructose were the main sugars present in the tip, while glucose was always consistently lower. Differences were also observed in absolute quantities, with total sugars in mid sections being around 10-fold higher compared to tip at harvest. Notably, sucrose concentrations in spear tips and mid-sections were similar in concentration at the beginning of storage (*ca*. 20 mg g^−1^ DW). Sucrose concentrations almost doubled by the end of shelf life irrespective of harvest time. (Supplementary material 5).

**Fig. 2:**
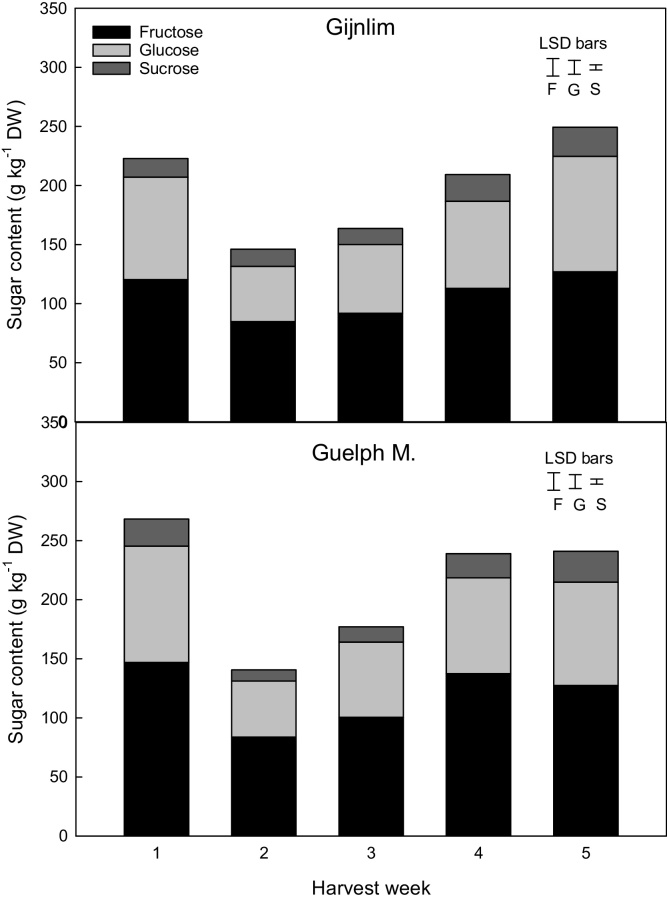
Seasonal changes in sugar content (fructose [F], glucose [G] and sucrose [S]) (mid-section) in two UK grown asparagus cultivars (‘Gijnlim’ and ‘Guelph M.’) measured across five consecutive harvest weeks. Each bar represents mean values of the three grades per cultivar. LSD bars (P *<* 0.05) shown were calculated using post-hoc Fisher test (for interaction between week*cultivar) for each individual sugar.

A significant seasonal effect was also observed in the sugar concentrations, with total sugars exhibiting a large dip on week two and week three with concentrations gradually increasing again by the end of the season. At the beginning of the season (week one) mid-sections of ‘Guelph M.’ spears had significantly higher (*ca.* 1.2 and 1.5-fold) fructose and sucrose contents compared to those of ‘Gijnlim’. Sugar concentrations were similar in both cultivars thereafter (Fig. 2) with the exception of week four when fructose and sucrose content was significantly higher in ‘Guelph M.’ compared to ‘Gijnlim’. Furthermore, ‘Guelph M.’ spears also had higher fructose and sucrose content in the tip region on week five (Supplementary material 6).

Sugar content varied only in the mid region between spears of different grades with larger grade spears having overall significantly higher sucrose content (21.0 g kg^−1^ DW) compared to small and medium spears (16.0 and 17.7 g kg^−1^ DW, respectively). In contrast, no significant differences in sucrose content in the tip region were observed between spears of different grades. Furthermore, glucose and fructose were similar irrespective of grade or spatial region.

A comparison between the percentages of individual sugar content against total sugars throughout the season revealed different trends between spatial regions in both cultivars. The percentage of sucrose in the mid region remained constant at ca. 7% of total sugars, irrespective of grade or cultivar. In contrast, for the tip, the ratio of sucrose increased during the season from 20% to 25% in ‘Gijnlim’, and from 23% to 35% for ‘Guelph M.’.

**Fig. 3:**
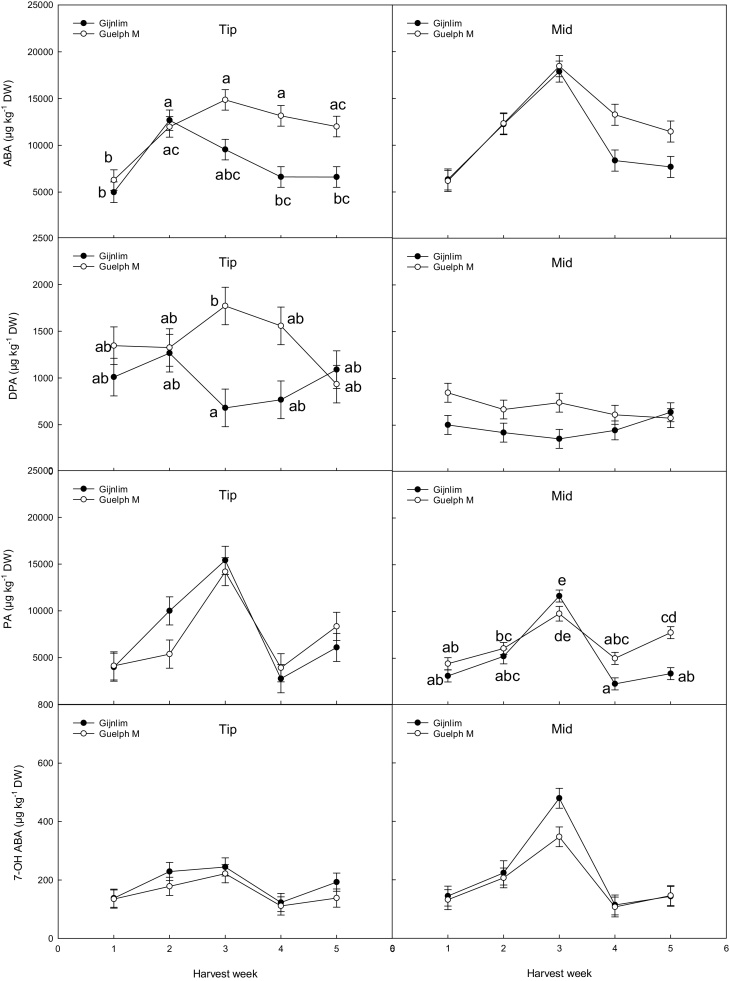
Seasonal changes in PGR content (μg kg^−1^ DW) (tip and mid-sections) in two UK grown asparagus cultivars (‘Gijnlim’ and ‘Guelph M.’) measured across five consecutive harvest weeks. Data represent a mean of three grades per cultivar (n = 9) ± standard deviation (SD). Different letters denote significant differences.

The seasonal variation of ABA and its catabolites was also measured for both cultivars. ABA, PA and 7−OH ABA contents increased in the mid region of the spear until week three and declined again thereafter (Fig. 3). ABA contents in the tip followed a similar trend in both cultivars up to week two with both showing a significant increase. However, while ABA in spears of ‘Guelph M.’ remained relatively stable thereafter, ‘Gijnlim’ spears gradually declined to initial concentrations observed early on in the season. Finally, DPA concentrations were overall higher in ‘Guelph M.’ compared to ‘Gijnlim’ in both the mid and tip sections.

A correlation was attempted between the mean weekly climatic conditions and the observed seasonal variation in the sugar and phytohormone profile in both cultivars using a generalised linear method with stepwise feature selection. The best results were obtained for total sugars and PA respectively in the apical section with good prediction accuracy (predicted vs observed values) (Supplementary material 7). In the case of total sugar content, the best predictors selected for the model were the mean radiation, RH, maximum daily temperature and average minimum daily temperature. Similarly, for PA the best predictors were mean RH, maximum daily temperature and average minimum daily temperature. Due to the relatively small size of the data set, these results can be viewed as preliminary and a more systematic sampling effort at shorter time intervals would be required in the future.

### 3.2 Temporal changes in physiology and biochemistry during cold storage and shelf life

In situ respiration rate (RR) was first measured 48 h after equilibration under cold (1 °C) conditions. Values steadily decreased during cold storage with RR highest in ‘Gijnlim’ (122.6 mg kg^−1^  h^−1^) on day two compared to ‘Guelph M.’ (57.1 mg kg^−1^ h^−1^); however after five days of storage, ‘Gijnlim’ samples exhibited a rapid *ca*. 80% decline in RR within the first week (Supplementary material 8). Contrastingly, RR of ‘Guelph M.’ samples remained constant throughout the storage period. Towards the end of cold storage (day 16), RR was significantly lower in ‘Jaleo’ and ‘Gijnlim’ compared to ‘Guelph M.’.

During cold storage, ‘Guelph M.’ (39.9 & 30.4) and ‘Gijnlim’ (39.0 & 30.0) maintained higher values for L* and C* compared to ‘Jaleo’ (49.1 & 36.9), respectively. Hue angle was similar between cultivars for the mid and base regions. However, for the tip, hue angle in ‘Gijnlim’ spears significantly dropped towards the end of cold storage and subsequent shelf life, with values 1.2-fold lower than the other two cultivars.

Stiffness significantly decreased during storage (from *ca*. 0.006 to 0.001 kHz kg^−1^) irrespective of cultivar; in contrast to mean cutting energy which steadily increased (from *ca*. 6–8.5 mJ) during three weeks of cold storage (+1 week shelf life) (data not shown). Mid-regions of ‘Jaleo’ spears had overall higher values for cutting energy (8.08 mJ) compared to those of ‘Gijnlim’ (6.20 mJ) and ‘Guelph M.’ (6.88 mJ). The same trend was observed for the apical regions with cutting energy for ‘Jaleo’ tips being up to 1.15-fold higher than ‘Gijnlim’ and ‘Guelph M.’ (data not shown).

**Fig. 4:**
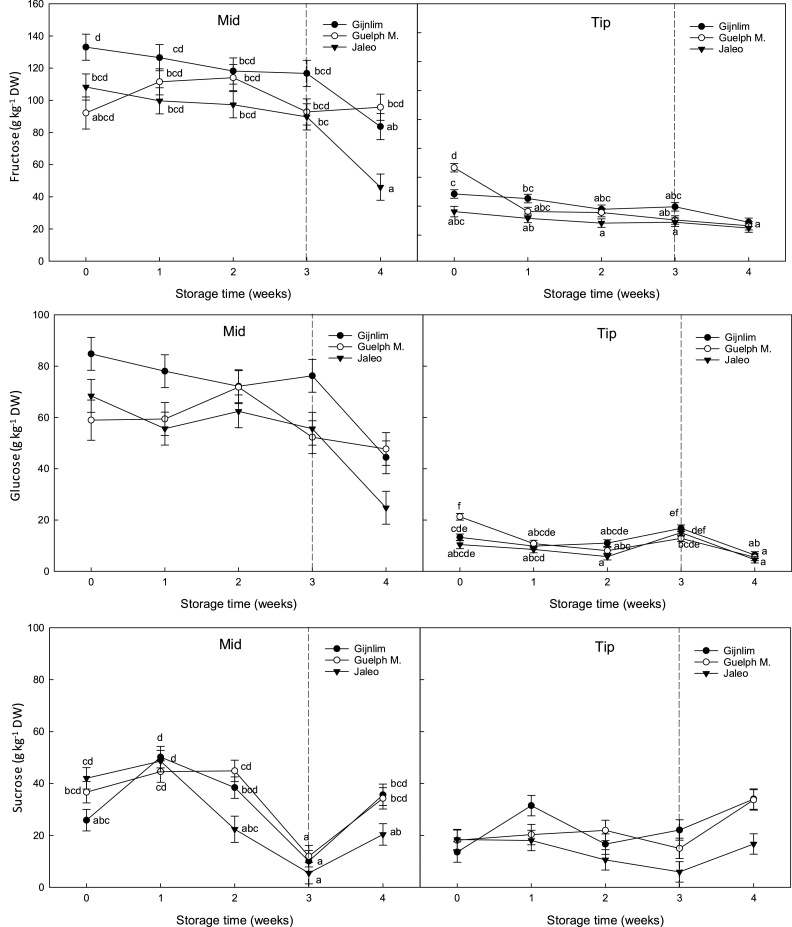
Temporal changes in sugar content (glucose, fructose and sucrose [g kg^−1^ DW]) in four asparagus cultivars (‘Gijnlim’, ‘Guelph M.’, and ‘Jaleo’) during cold storage at 1 °C for 3 weeks followed by shelf life assessment at 7 °C for 7 d. Data represent means (n = 3) ± standard deviation (SD). Different letters denote significant differences.

During cold storage, sugar concentrations steadily decreased (Fig. 4). At week two of storage, sucrose contents in the mid region of ‘Jaleo’ and overall apical sucrose content (13.9 g kg^−1^ DW) were significantly lower than in samples of the other two cultivars. Furthermore, ‘Jaleo’ spears experienced a significant decrease in fructose in the mid-section during shelf life (start indicated by dotted line on graphs), with values falling to ca. 50 g kg^-1^ DW, which was significantly lower compared to the other two cultivars (Fig. 4). This trend was not significant for glucose. In contrast, sucrose in the tip and mid regions significantly increased more than two-fold during shelf life, irrespective of cultivar. This increase in sucrose also occurred during shelf life over five consecutive weeks.

Total ascorbic acid steadily increased during cold storage in both the mid and tip sections; but the greatest increase was more evident in the tip with average values increasing from 4.2 up to 7.5 g kg^−1^ DW, irrespective of cultivar (Supplementary material 9). An overall cultivar effect was observed in the tip where ‘Jaleo’ spears contained the highest ascorbic acid concentration (5.88 g kg^−1^ DW) compared to ‘Gijnlim’ (4.92 g kg^−1^ DW) and ‘Guelph M.’ (4.86 g kg^−1^ DW).

## 4 Discussion

### 4.1 Seasonal changes and genotype affect storage potential

Fluctuations in temperature during the harvest season (Supplementary material 10) appeared to coincide with changes in spear physiology and biochemical profile. The peak in sugar and ascorbic acid in both cultivars observed at week one coincided with cooler temperatures prior to harvest (11 °C max and 5 °C min). These results are in agreement with that described previously by Bhowmik et al. (2002) and Shou et al. (2007) where carbohydrate content in spears was highest during cooler temperatures; and Smirnoff (2018) who reported low temperatures induced ascorbic acid accumulation in asparagus spears. Low temperatures can induce abiotic stress resulting in accumulation of reactive oxygen species (ROS) causing in turn an increase in ascorbic acid biosynthesis in an attempt to protect the plant from oxidative stress (Conklin, 2001).

The subsequent drop in sugar and ascorbic acid contents may reflect the effect of the more favourable growing conditions. These assumptions were further supported by the good correlation obtained between solar radiation and total sugar content based on the acquired prediction model. Indeed, a rapid depletion of sugars has been reported in actively growing acropetal tissues as a result of high RR which was up to 4 times higher compared to basal regions (Verlinden et al., 2014). Previous studies have also shown higher sugar content in large diameter (slower growing) spears, compared to small diameter (faster growing) spears (Hurst et al., 1993). The steady increase in temperatures after week one, in combination with more solar radiation exposure (Supplementary material 11), are likely to have promoted growth, coinciding with reduced fibre content, thus causing the observed decrease in cutting energy and stiffness values. An increase in asparagus shear force is attributed to cooler temperatures resulting in slower growth (Bhowmik et al., 2002; Lipton, 1990). Furthermore, the lower hue angle values observed in all spatial regions at week one could have been attributed to differences in chlorophyll, flavonoid (*viz*. rutin) and or anthocyanin content present on the skin, which are influenced by sunlight exposure (Siomos et al., 2001; Wambrauw et al., 2016).

### 4.2 Seasonal variation of ABA and catabolites

ABA and its main catabolite PA, exhibited the opposite trend to sugars reaching maximum levels towards the middle of the season. ABA has been proposed to play an active role in promoting spear growth and sugar mobilisation from the crown to the spear with increased amounts reportedly observed in lateral buds and the tip of the spear during the elongation phase with a decreasing spatial distribution along the length of the spears (Kojima et al., 1993). Similarly, the highest levels of ABA in this study were observed during favourable growing conditions, although there were no major differences observed in the spatial distribution between apical and mid sections. ABA and ABA catabolite accumulation may also have been driven by water stress due to a combination of warm and dry conditions and increased RR. RH was among the best predictors of PA content in asparagus tips, which may be linked to ABA (and catabolites) accumulation under low RH conditions and increased temperatures to prevent excessive water loss. The subsequent drop in ABA and sugar levels towards the end of the season may have been due to a combination of increased rainfall and a reduction in yield as the season progressed. Indeed, over time, the lag time in growth between spears on the same cluster increases, and the number of viable buds declines resulting in a gradual decrease in spear production (Feller et al., 2012). This phenomenon was more pronounced for ‘Gijnlim’, compared to ‘Guelph M.’ with the latter also maintaining higher ABA levels past week three, which could be attributed to the cultivar profile, since ‘Guelph M.’ is a late maturing variety (in comparison to ‘Gijnlim’) and is used to extend the UK season.

### 4.3 Seasonal variation in carbohydrate reserves

Season progression has been associated with a decline in spear diameter (Hurst et al., 1993) and sugar content (Bhowmik et al., 2002), which has been attributed to storage root carbohydrate depletion over time. To support metabolic activity in the tip, sugars in the mid region of the spear are exported to the acropetal regions (Verlinden et al., 2014), which may explain the shift in sugar ratios observed in the apical sections of the spears towards the end of the season. These observed variations in sugar ratios may affect perceived taste since individual sugars have different sweetness profiles (Mahawanich and Schmidt, 2004).

### 4.4 Carbohydrate metabolism during storage

Carbohydrate content in asparagus spears did not decline under shelf life conditions (7 °C under light). This finding and in particular the increase in sucrose, contradicts the majority of literature which generally reports a steady decline in sugars (Hurst et al., 1993; Bhowmik et al., 2002; Verlinden et al., 2014), with the loss of sucrose thought to initiate the cascade of events leading to spear deterioration (Renquist et al., 2005). However, in these previous studies, shelf life temperatures were set above 20 °C (as opposed to 7 °C) or under very cold conditions (0 °C) which are not reflective of conditions commonly found in the supply chain. Therefore, differences in temperature could have a significant impact on how the sugar profiles change during storage. One previous study also reported an increase in spear sucrose during storage (7 d) under different temperatures (ranging from 0.5–20 °C) whilst kept under saturated moisture conditions, with the highest increase observed at 5 and 10 °C (Herppich and Huyskens-Keil, 2008), which is in good agreement with the findings herein.

Notably, the postharvest shift in sucrose synthesis only occurred under shelf life conditions (7 °C). In contrast, during cold storage (1 °C under dark conditions) sugars remained relatively stable with the exception of sucrose (mid sections), which showed a steep decline at the end of cold storage. However, once samples were transferred and held under shelf life conditions (7 °C under light conditions), reducing sugars rapidly declined while sucrose increased suggesting a condensation reaction. This reaction is catalysed by sucrose phosphate synthase (SPS) which follows a circadian pattern of activity (Jones and Ort, 1997). The differentiation in sugar profiles during cold storage and shelf life could therefore be linked to variations in light exposure and temperature.

Cultivar differences may also be a significant factor in affecting sugar metabolism and spear quality postharvest. Cultivar ‘Jaleo’ showed a much more rapid decrease in reducing sugars, and only a moderate increase in sucrose concentrations compared to the other two cultivars; while apical regions of spears ‘Gijnlim’ had a significant drop in hue angle indicative of a rapid loss of chlorophyll and thus yellowing. These results could be attributed to a combination of higher RR at the beginning of storage, resulting in more rapid consumption of non-structural carbohydrate reserves leading to a faster rate of senescence.

### 4.5 Textural changes during storage

In general, spears ‘Guelph M.’ retained better quality by the end of cold storage and subsequent shelf life compared to the other two cultivars, characterised by high sugar levels and stable colour. Nevertheless, storage time had a negative impact on spear texture irrespective of cultivar, with cutting energy increasing and stiffness decreasing both during cold storage and subsequent shelf life. This is indicative of lignification processes taking place and the thickening of secondary cell walls (mainly sclerenchyma sheath cells and vascular bundle elements) leading to an increase in cutting energy (Herppich and Huyskens-Keil, 2008), while simultaneously moisture loss contributed to the decrease in stiffness as measured using laser Doppler vibrometry. In our work, a poor correlation (r^2^ = 0.21) between stiffness (whole spear) and cutting energy (at either tip or mid region [mJ]) was observed possibly due to the spatial variation in cutting energy along the spear. Others have also reported a weak correlation between stiffness and cutting energy in other crops, and this was associated with various factors including heterogeneity of the sample (Shmulevich et al., 2003; Molina-Delgado et al., 2009; Cui et al., 2015). Consequently, both methods can provide different insights into the textural changes occurring in asparagus during storage.

## 5 Conclusion

The seasonal variation in the physiological and biochemical profile (sugars and ABA in particular) recorded over five weeks was shown to have a direct impact on the storage potential during shelf life conditions. A significant shift in sugar biosynthesis under shelf life conditions is reported here promoting the condensation reaction of reducing sugars (glucose and fructose) to form sucrose. The trigger for the shift in sugar metabolism is unclear but is likely to be linked to differences in temperature, circadian controls and light exposure during storage. Additional work is required to confirm this hypothesis. Genotype was also a contributing factor influencing the rate of metabolic changes during storage, with late maturing cultivars exhibiting a slower senescence rate.

All authors have participated in (a) conception and design, or analysis and interpretation of the data; (b) drafting the article or revising it critically for important intellectual content; and (c) approval of the final version.

## Declaration of Competing Interest

All authors have participated in (a) conception and design, or analysis and interpretation of the data; (b) drafting the article or revising it critically for important intellectual content; and (c) approval of the final version.

This manuscript has not been submitted to, nor is under review at, another journal or other publishing venue.

The authors have no affiliation with any organization with a direct or indirect financial interest in the subject matter discussed in the manuscript.
