# Supplementary material for: Seasonal and temporal changes during storage affect quality attributes of green asparagus
Source: Postharvest Biol Technol. 2020 Jan;159:111017. doi: 10.1016/j.postharvbio.2019.111017 (PMC6853415; doi:10.1016/j.postharvbio.2019.111017)
Supplement: Supplementary file 1 [file mmc1.docx]

**Supplementary material**

**Supplementary material 1.** Seasonal changes in colour (L*, C* and h°) in two UK grown asparagus cultivars (‘Gijnlim’ and ‘Guelph M.’) measured across five consecutive harvest weeks starting from the 25^th^ May to 23^rd^ June 2016. Data represent a mean of three grades per cultivar (n=9) ± standard deviation (SD). Different letters denote significant differences for the base (11-15 cm), mid (4-11 cm) and tip (0-4 cm) region of asparagus spears.

| **Harvest week** | **Sample day** | **Gijnlim** | **Guelph M.** |
| --- | --- | --- | --- |
| 1 | 0 | 6.70 | 7.69 |
| 1 | 2 | 5.44 | 7.46 |
| 1 | 5 | 6.89 | 8.84 |
| 2 | 0 | 4.82 | 5.27 |
| 2 | 2 | 6.03 | 7.87 |
| 2 | 5 | 7.26 | 7.74 |
| 3 | 0 | 5.23 | 5.20 |
| 3 | 2 | 4.88 | 5.17 |
| 3 | 5 | 4.94 | 5.40 |
| 4 | 0 | 4.16 | 5.06 |
| 4 | 2 | 4.58 | 4.65 |
| 4 | 5 | 4.37 | 5.66 |
| 5 | 0 | 4.55 | 5.64 |
| 5 | 2 | 4.36 | 5.33 |
| 5 | 5 | 5.63 | 5.44 |

**Supplementary material 2**, Seasonal changes in cutting energy (mJ) for both cultivars, measured during shelf life across five consecutive harvest weeks starting from the 25^th^ May to 23^rd^ June 2016. Data represent a mean of three grades (n=9). LSD = 0.87 (P < 0.05).

b

a

**Supplementary material 3.** Seasonal changes in overall [a] cutting energy (mJ) [b] stiffness (kHz Kg^-1^) (for mid [at 11cm from top] and tip [at 4 cm from top] region of asparagus spears), as a mean for both cultivars, measured across five consecutive harvest weeks starting from the 25^th^ May to 23^rd^ June 2016. Data for cutting energy represent a mean of two cultivars and three grades (n=18) while data for stiffness represent a mean of two cultivars, three grades and two sections (n=36) ± standard deviation (SD). Significant differences denoted by different letters for each graph.

**Supplementary material 4**, Seasonal changes in moisture loss (%) for both cultivars, measured during shelf life across five consecutive harvest weeks starting from the 25^th^ May to 23^rd^ June 2016. Data represent a mean of three grades (n=9). Significant differences denoted by different letters for each graph. LSD = 1.4 (P < 0.05).

| **Harvest week** | **Grade** | **Sampling day** | **Moisture loss (%)** | |
| --- | --- | --- | --- | --- |
|  |  |  | **Gijnlim** | **Guelph M.** |
| 1 | L | 2 | 2.0 | 2.4 |
| 1 | L | 5 | 3.0 | 4.1 |
| 1 | M | 2 | 2.7 | 2.5 |
| 1 | M | 5 | 3.5 | 4.9 |
| 1 | S | 2 | 3.7 | 4.5 |
| 1 | S | 5 | 7.5 | 6.1 |
| 2 | L | 2 | 1.6 | 2.8 |
| 2 | L | 5 | 3.4 | 4.1 |
| 2 | M | 2 | 1.8 | 2.8 |
| 2 | M | 5 | 5.2 | 3.7 |
| 2 | S | 2 | 6.6 | 4.7 |
| 2 | S | 5 | 8.9 | 7.8 |
| 3 | L | 2 | 1.2 | 1.3 |
| 3 | L | 5 | 4.1 | 5.2 |
| 3 | M | 2 | 1.2 | 0.9 |
| 3 | M | 5 | 5.4 | 5.5 |
| 3 | S | 2 | 1.3 | 2.6 |
| 3 | S | 5 | 6.0 | 6.9 |
| 4 | L | 2 | 2.3 | 2.9 |
| 4 | L | 5 | 4.6 | 5.9 |
| 4 | M | 2 | 1.4 | 3.6 |
| 4 | M | 5 | 4.2 | 6.5 |
| 4 | S | 2 | 3.3 | 3.0 |
| 4 | S | 5 | 7.6 | 10.7 |
| 5 | L | 2 | 2.1 | 1.8 |
| 5 | L | 5 | 4.4 | 6.9 |
| 5 | M | 2 | 4.5 | 2.8 |
| 5 | M | 5 | 5.3 | 8.6 |
| 5 | S | 2 | 6.4 | 6.0 |
| 5 | S | 5 | 9.2 | 13.2 |

**Supplementary material 5.** Temporal changes in sucrose content (g kg^-1^ DW) for tip and mid region of asparagus spears in two medium grade UK grown cultivars (‘Gijnlim’ and ‘Guelph M.’) during 5 days shelf life storage measured across five consecutive harvest weeks starting from the 25^th^ May to 23^rd^ June 2016. Data represent means (n=3) ± standard deviation (SD). Different letters within each graph denote significant differences.

**Supplementary material 6**. Seasonal changes in sugar content (fructose, glucose and sucrose [g kg^-1^ DW]) for tip regions (0-4 cm) in two UK grown asparagus cultivars (‘Gijnlim’ and ‘Guelph M.’) measured across five consecutive harvest weeks starting from the 25^th^ May to 23^rd^ June 2016. Data represent a mean of three grades per cultivar (n=9) ± standard deviation (SD). Different letters within each graph denote significant differences.


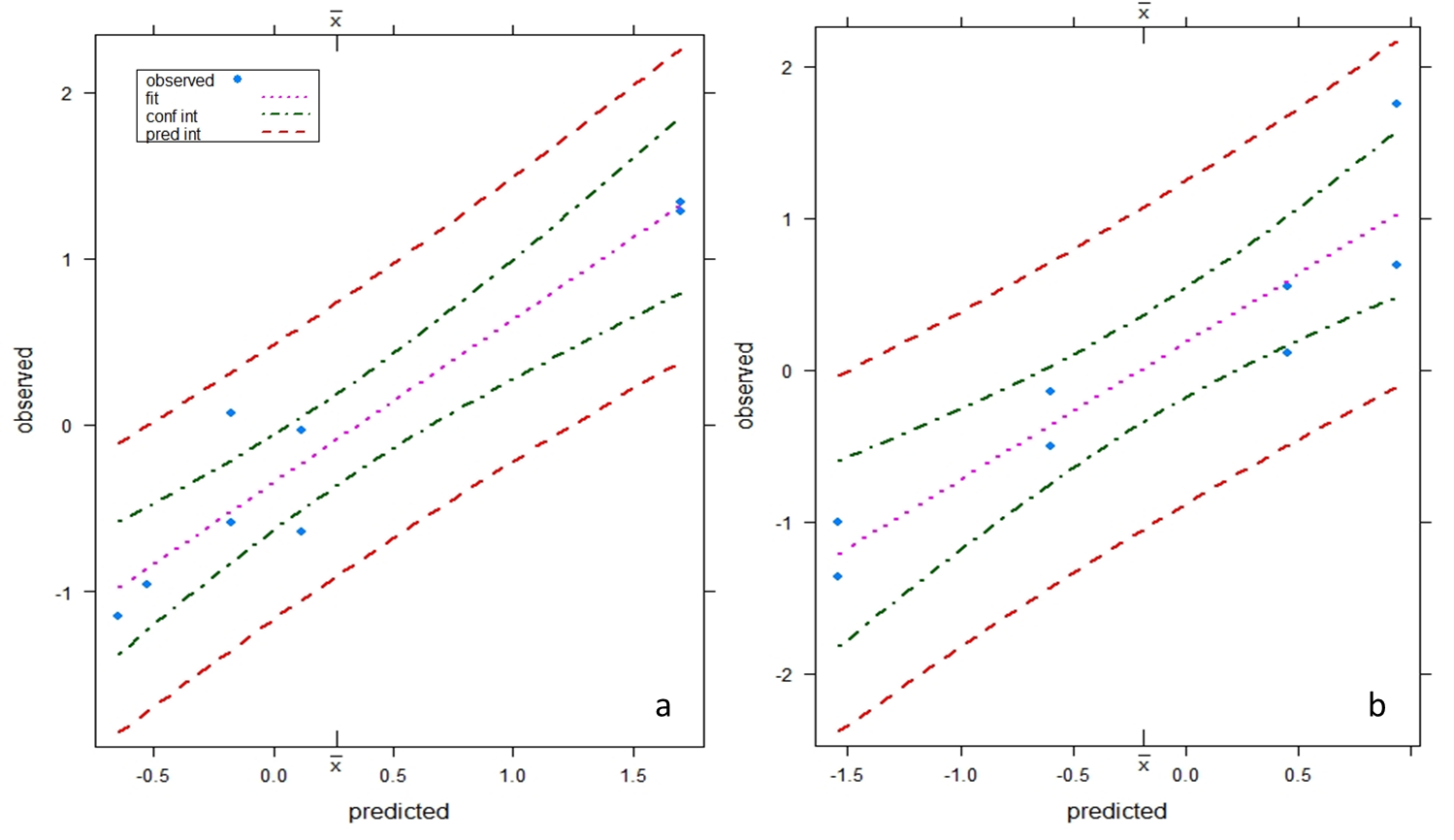


**Supplementary material 7**, Plot comparing mean weekly climatic conditions (including max. daily temp + min daily temp + relative humidity + radiation) and observed seasonal variation in phaseic acid (PA) content [a], and total sugar content [b] against predicted values (with 95% confidence interval) using glm stepwise AIC model. RMSE = 0.445 and 0.417 for PA and sugar, respectively.

**Supplementary 8.** Temporal changes in respiration rate (mg kg^-1^ h^-1^) in three asparagus cultivars (‘Gijnlim’, ‘Guelph M.’ and ‘Jaleo’) during cold storage at 1 °C for 3 weeks. Data represent means (n=3) ± standard deviation (SD). Different letters denote significant variations.

**Supplementary material 9**. Temporal changes in ascorbic acid (g kg^-1^ DW) for mid [11 - 4cm] and tip [0 - 4 cm] region of asparagus spears in three cultivars (‘Gijnlim’, ‘Guelph M.’ and ‘Jaleo’) during cold storage at 1 °C for 3 weeks followed by shelf life assessment at 7 °C for 7 days. Data represent means (n=3) ± standard deviation (SD). Different letters denote significant variations.

**Supplementary material 10.** Weather data (*viz*. min and max temperature (°C), and rainfall [mm]) from the start of the harvest season (middle of May) to end of harvest (late June 2016). Data was obtained from an independent weather station (coordinates: 51.894189,-2.560535) located at Cobrey Farms (Ross-on-Wye, UK). Dashed vertical lines indicate the harvest date for the five asparagus batches received at Cranfield (weeks 1 to 5).

**Supplementary material 11**. (a) Maximum radiation levels (W m^2^), (b) RH (%) and wind speed (km h^-1^) during the harvest season 2016. Weather data was obtained from an independent weather station (coordinates: 51.894189,-2.560535) located at Cobrey Farms (Ross-on-Wye, UK). Dashed vertical lines indicate the harvest date for the five asparagus batches received at Cranfield (weeks 1 to 5).
